# *Zic1* Promoter Hypermethylation in Plasma DNA Is a Potential Biomarker for Gastric Cancer and Intraepithelial Neoplasia

**DOI:** 10.1371/journal.pone.0133906

**Published:** 2015-07-24

**Authors:** Xueqin Chen, Zhenghua Lin, Meng Xue, Jianmin Si, Shujie Chen

**Affiliations:** 1 Department of Gastroenterology, Sir Run Run Shaw Hospital, Zhejiang University, Hangzhou, Zhejiang, China; 2 Institute of Gastroenterology, Zhejiang University, Hangzhou, Zhejiang, China; Sapporo Medical University, JAPAN

## Abstract

Gastric cancer (GC) remains one of the most common digestive cancers worldwide; however, most patients present at an advanced stage at initial diagnosis. *Zic1* is a novel candidate tumor suppressor gene that is epigenetically silenced in GC. In this study, we investigated *Zic1* promoter methylation in plasma DNA as a novel molecular marker for the early diagnosis and monitoring of GC. Methylation-specific polymerase chain reaction (MSP) assay was performed to detect *Zic1* promoter methylation in plasma DNA from 20 healthy subjects, 50 gastric intraepithelial neoplasia patients, and 104 GC patients. The *Zic1* promoter methylation rate in the plasma samples from the healthy control group was 0%, but it reached 54.0% in the intraepithelial neoplasia group and 60.6% in the GC group. The latter two values were significantly higher than that found in the healthy control group (p < 0.05), with a 100% specificity for intraepithelial neoplasia and GC diagnosis. The positive predictive value of plasma *Zic1* promoter methylation for the diagnosis of intraepithelial neoplasia and GC was 100%. Methylation status in the GC group was not significantly associated with tumor size, tumor differentiation, lymph node metastasis, TNM staging, or tumor invasion (p > 0.05). Assessment of the significance of detection of the carcino-embryonic antigen (CEA) level and *Zic1* promoter methylation rate for GC diagnosis revealed that the sensitivity of *Zic1* promoter methylation was significantly higher than that of the CEA level as a marker and that the combined measurement of these two indices (parallel testing) improved sensitivity. Taken together, our results suggest that the *Zic1* promoter methylation rate in plasma-derived DNA is of great significance for the early screening of GC and monitoring of tumorigenesis. *Zic1* promoter methylation may serve as a novel non-invasive plasma biomarker for the early detection of GC and for risk assessment in high-risk populations. The combined measurement of the *Zic1* promoter methylation rate and CEA level (parallel testing) may enhance the current guidelines for the early diagnosis of GC.

## Introduction

Gastric cancer (GC) is one of the most common digestive cancers and the third leading cause of cancer-related deaths worldwide[1]. The majority of GC patients present at an advanced stage upon initial diagnosis. Globally, GC accounts for nearly 952,000 new cases annually according to Globocan 2012[2]. In Japan, the 5-year survival rate for early GC (EGC) is 86% compared with 32% for advanced GC, as reported by the US SEER program[3].

Aberrant DNA methylation is an important epigenetic change and an early event in carcinogenesis[4]. Recently, several tumor suppressor genes (TSGs), including *FAM5C*, *MYLK*, *p16* and *E-cadherin*, have been reported to be methylated in the serum/plasma of GC patients[5–7]. In addition, hypermethylation of TSGs tends to be enhanced with the progression of pre-cancerous lesions[8–9]. Therefore, the detection of methylated DNA in serum/plasma may be one promising approach for the early diagnosis of GC. Intraepithelial neoplasia (IN) is a potentially precancerous lesion that confers a high risk of GC; thus, the screening of IN patients is crucial for diagnosis and prediction of GC. Few reports of hypermethylated DNA analyses of blood samples from gastric intraepithelial neoplasia (GIN) patients are available to date.

Growing evidence indicates that *Zic1* (*Zic* family member 1, odd-paired Drosophila homolog) participates in the progression of several cancers[10–13]. We have previously discovered that *Zic1*, a novel TSG, is silenced via promoter hypermethylation in both GC cells and tissues[14]. We have also demonstrated that *Zic1* is involved in inhibition of GC cell survival and impairment of cell migration[15].

In the present study, we determined the early diagnostic and predictive potentials of *Zic1* hypermethylation in the plasma of GC patients. We measured the promoter methylation status of *Zic1* in plasma from patients with GC or GIN and normal control (NC) subjects. The association of *Zic1* promoter methylation with clinicopathological parameters was also assessed. In addition, we classified high-grade IN and T1 GC as EGC and placed the GC and GIN patients in a GC plus IN group (GPI). Then, we analyzed the *Zic1* hypermethylation rates in both the EGC and GPI groups. Further, the combined evaluation of the carcino-embryonic antigen (CEA) level and *Zic1* promoter methylation for GC diagnosis was assessed using plasma from the GC and GIN patients.

## Materials and Methods

### Clinical samples

Plasma samples were obtained preoperatively at Sir Run Run Shaw Hospital, Hangzhou, Zhejiang, China, from November 2012 to April 2015. All of the patients included in our study underwent gastroendoscopy and were grouped according to histopathology diagnosis. Plasma samples were collected from GC and GIN patients who were naïve to curative treatments, such as chemotherapy, radiotherapy, and surgical resection. The exclusion criteria included gastrointestinal diseases with other causes and diseases in other organs. Patients with gastric metastases from other tumors were also excluded.

We numbered the samples according to the order in which they were collected and then performed methylation-specific polymerase chain reaction (MSP) to assess the samples in numerical order. The samples were further grouped according to the pathology results. Plasma samples were collected from 104 GC patients with an average age of 59.8 years who were diagnosed according to the World Health Organization criteria[16]. Fifty GIN patients with an average age of 58.0 years were diagnosed by gastroscopy. Blood samples were also collected from 20 healthy volunteers in the NC group with an average age of 38.5 years.

### Ethics statement

Our project received approval from the Clinical Research Ethics Committee of the Institute of Gastroenterology of Zhejiang University. Written informed consent was obtained from each patient before sample collection. The individuals who participated in this study gave written informed consent (as outlined in the *PLOS* consent form) to publish these case details.

### DNA isolation and bisulfite modification

DNA was extracted from the plasma samples using a QIAamp DNA Blood Mini Kit (Qiagen, Hilden, Germany). DNA concentrations were measured by spectroscopy at a wavelength of 260 nm. DNA was bisulfite-treated with a Zymo DNA Modification Kit (Zymo Research, Orange, CA, USA). The modified DNA was stored at -20°C until use.

### MSP assays

*Zic1* promoter methylation was examined by performing MSP assay using the above mentioned bisulfite-modified free plasma DNA as the template. MSP was carried out for 40 cycles at an annealing temperature of 56°C as described previously[14]. The methylation-specific primers were ZIC1-MF (5’-GGATTTTTTGTTTCGTAATC) and ZIC1-MR (5’-CCCGTTAACCACGTTAAACG), and the unmethylated-specific primers were ZIC1-UF (5’-GGGATTTTTTGTTTTGTAATT) and ZIC1-UR (5’-CCCATTAACCACATTAAACA). The PCR protocol included initial denaturation at 95°C for 10 min, followed by 40 cycles of denaturation at 95°C for 30 s, annealing at 56°C for 40 s, primer extension at 72°C for 40 s, and final extension at 72°C for 7 min. The PCR products were electrophoresed on 1.5% agarose gels, stained with Gel Red, and visualized by UV illumination.

### Serum CEA testing

The serum samples were sent to the clinical laboratory of Sir Run Run Shaw Hospital for testing, and a CEA concentration of > 5 ng/mL was regarded as abnormal.

### Statistical analyses

The methylation frequencies and CEA levels in the plasma samples were compared using Fisher's exact test. The association between the clinicopathological characteristics and DNA hypermethylation was analyzed using the chi-square test. The diagnostic value of the combination of *Zic1* promoter methylation and the plasma CEA level was evaluated according to the area under the receiver operating characteristic curve (AUC). The sensitivity, specificity, and positive and negative predictive values for pathological diagnosis were calculated with 95% confidence intervals for *Zic1* promoter methylation or *Zic1* promoter methylation combined with the CEA level. Positive and negative likelihood ratios were also calculated. For all of the tests, a cut-off value of p < 0.05 was applied to determine statistical significance. All statistical analyses were conducted using SPSS 19.0 software (SPSS Inc., Chicago, IL).

## Results

### Methylation status in the plasma

**Fig 1:**
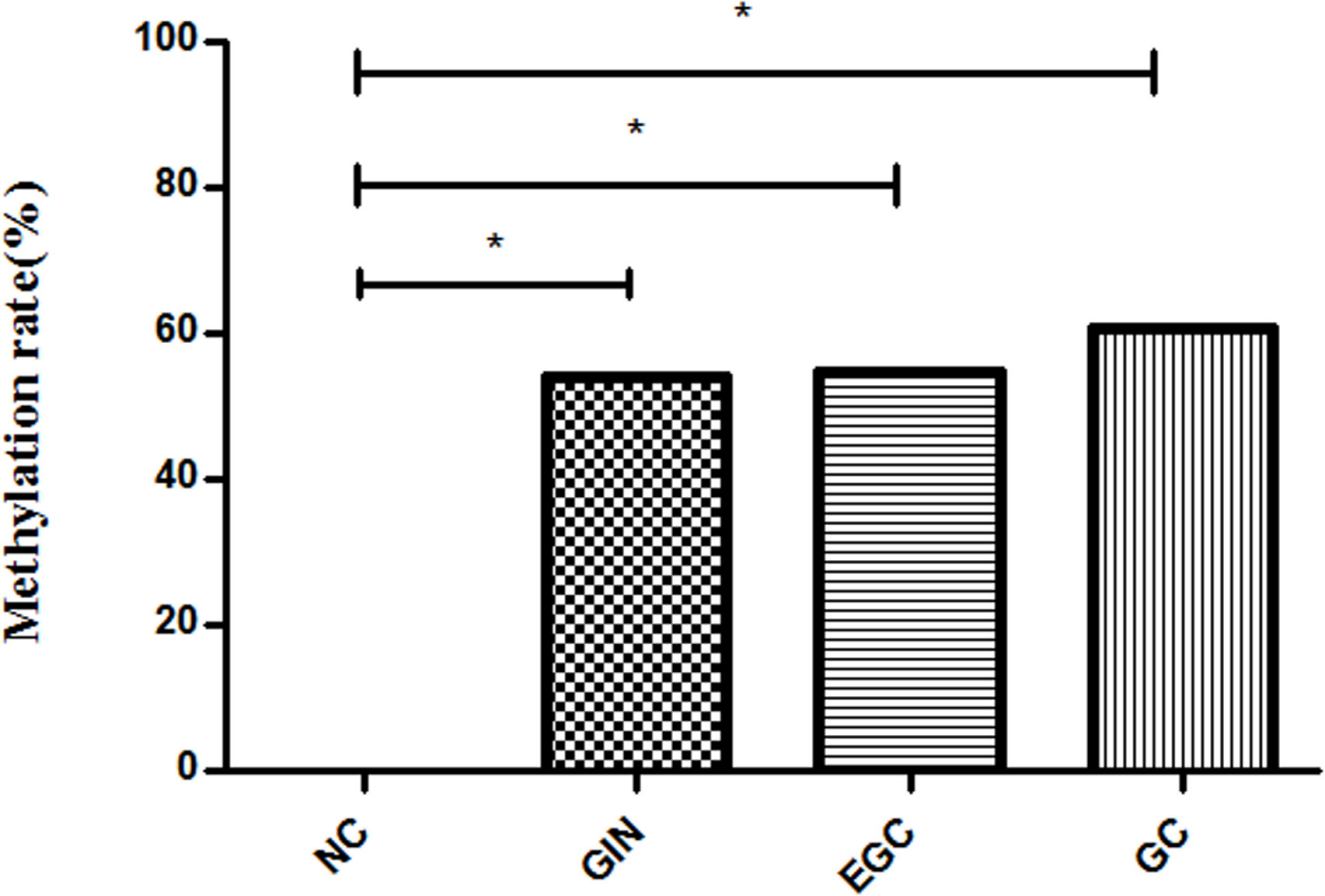
Percentage of *Zic1* promoter methylation in the gastric cancer (GC), gastric intraepithelial neoplasia (GIN), early gastric cancer (ECG) and normal control (NC) groups. The percentages of *Zic1* promoter methylation were 60.6% (63/104) in the GC, 54.0% (27/50) in the GIN, 54.8% (17/31) in the EGC and 0.0% (0/20) in the NC groups (*: p < 0.001).

**Fig 2:**
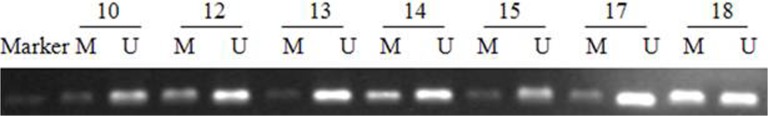
Representative *Zic1* promoter methylation, as determined by methylation-specific polymerase chain reaction (MSP) assay. M: Methylation-specific primers; U: Unmethylated-specific primers.

To assess alterations in *Zic1* promoter methylation in GC, we first detected the methylation status of this promoter in 104 patients with GC, 50 with GIN, 31 with EGC and 20 NCs and performed comparisons among the groups. The methylation rates of the *Zic1* promoter in the plasma samples were 0% in the NC group (0%), increasing to 54.0% in the GIN group and to 60.6% in the GC group (p < 0.001 for both GIN and GC vs. NC), with 100% specificity for GPI diagnosis (20/20) (Fig 1). The positive and negative predictive values and positive and negative likelihood ratios for diagnosing IN and GC were 100% (90/90), 23.8% (20/84), infinite and 0.42, respectively. Representative MSP assay results for *Zic1* promoter methylation are shown in Fig 2.

### Association of clinicopathological features with *Zic1* promoter methylation in the plasma of GC patients

We then correlated the clinicopathological features with the *Zic1* promoter methylation rate in the plasma of the GC patients to identify any associations. The results revealed no marked associations between the *Zic1* promoter methylation rate and the clinical parameters, including tumor size, differentiation grade, lymph node status, tumor invasion depth and TNM stage (p > 0.05) (Table 1).

**Table 1:** Clinicopathological parameters and detection of *Zic1* hypermethylation in plasma from gastric cancer (GC) patients.

Characteristics		*Zic1*			
	n	M	U	χ2	p
Tumor size					
≥ 5 cm	31	21	10		
< 5 cm	53	29	24	1.377	0.260
Differentiation grade					
low	52	33	19		
medium / high	24	14	10	0.083	0.546
Lymph node status					
N0	35	19	16		
N1-3	48	31	17	0.896	0.372
Tumor invasion depth					
T1/T2	28	15	13		
T3/T4	56	36	20	0.898	0.355
TNM Stage					
0-II	31	19	12		
III-IV	45	27	18	0.013	1.000

M: Methylated; U: Unmethylated

### Combined determination of *Zic1* hypermethylation and CEA level

The significance of the combined determination of the *Zic1* hypermethylation rate and the CEA level for the diagnosis and early prediction of GC was analyzed. We measured the CEA level in 9 NCs, 24 GIN patients and 71 GC patients before treatment. The sensitivities of the CEA level in the GC, IN, and EGC groups were 22.5% (16/71), 16.7% (4/24) and 10.5% (2/19), respectively. In all 3 of the groups, the sensitivity of *Zic1* promoter methylation was significantly higher than that of the CEA level (p < 0.05).

#### Combined determination of *Zic1* hypermethylation and CEA level in GC group

**Fig 3:**
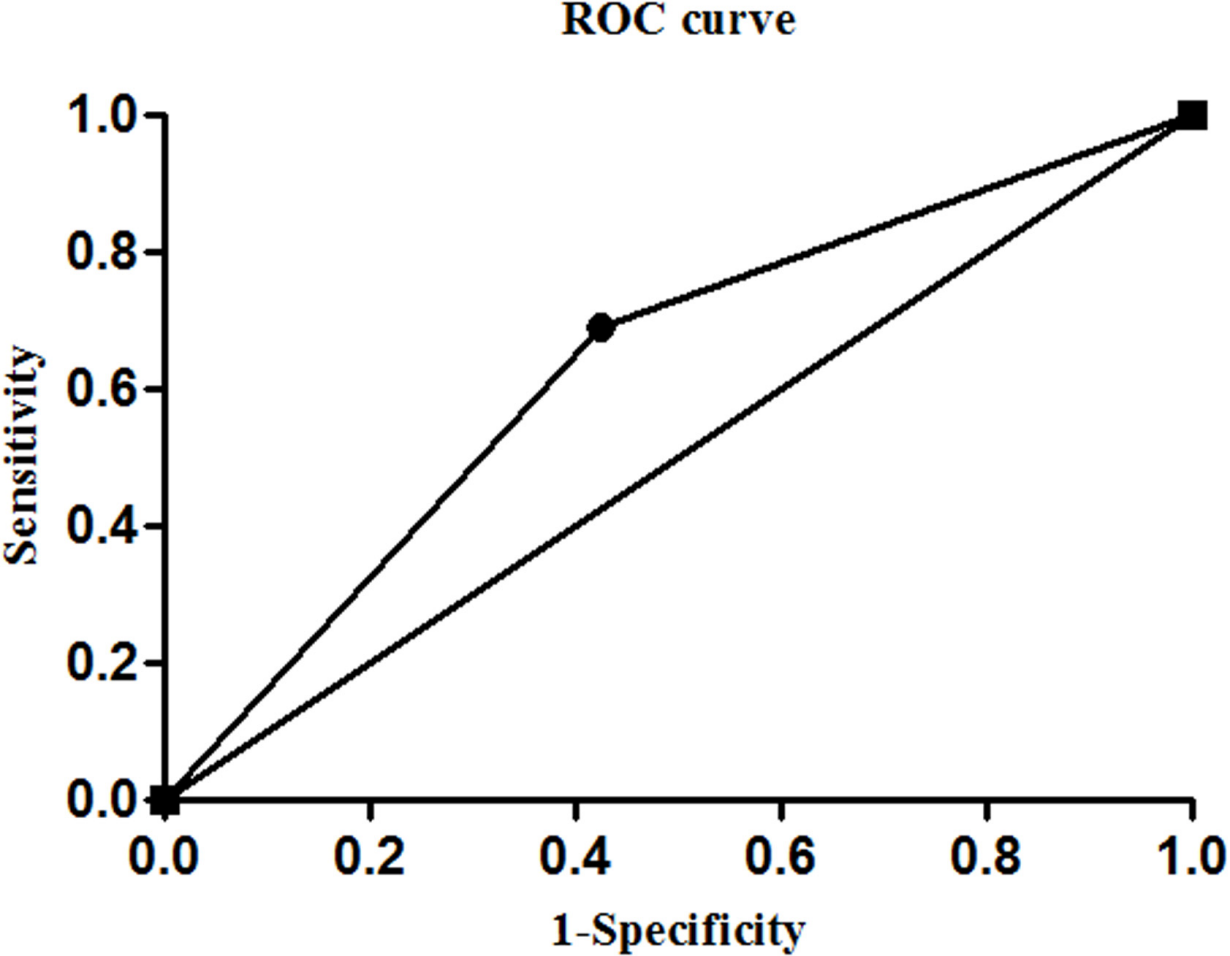
Combined detection of *Zic1* promoter methylation and the CEA level (parallel testing) in gastric cancer (GC) plasma specimens. An ROC curve for evaluating the significance of the combined detection of the two parameters for GC diagnosis.

In the GC group, the sensitivity and specificity of the combined determination of the *Zic1* promoter methylation rate and the CEA level (tandem testing) were 16.9% and 90.9%, respectively (Table 2). The sensitivity of the combined analysis of these two parameters (parallel testing) was 69.0% (Table 2), which was higher than the sensitivities achieved by the testing of either parameter alone. ROC curve analysis was performed to assess the significance of the DNA methylation rate and CEA level for GC diagnosis. The AUC for *Zic1* promoter methylation testing alone was 0.610 (S1 Fig) and that for the combined assessment of *Zic1* promoter methylation and the CEA level (tandem testing) was 0.539 (S2 Fig). Notably, when *Zic1* promoter methylation and the CEA level were assayed in parallel, the AUC increased to 0.633 (Fig 3). Based on the above results, the combined determination of the *Zic1* promoter methylation rate and CEA level (parallel testing) for GC diagnosis was found to be synergistic compared with the use of each method alone.

**Table 2:** Sensitivity and specificity of markers detected in the gastric cancer (GC) group.

Marker	Sensitivity (%)	Specificity (%)
CEA	22.5%	84.8%
*Zic1* promoter methylation	60.6%	61.4%
CEA* *Zic1* promoter methylation	16.9%	90.9%
CEA/ *Zic1* promoter methylation	69.0%	57.6%

*: *Zic1* promoter methylation combined with the CEA level (tandem testing)

/: *Zic1* promoter methylation combined with the CEA level (parallel testing)

#### Combined determination of *Zic1* hypermethylation and CEA level in GPI group

**Fig 4:**
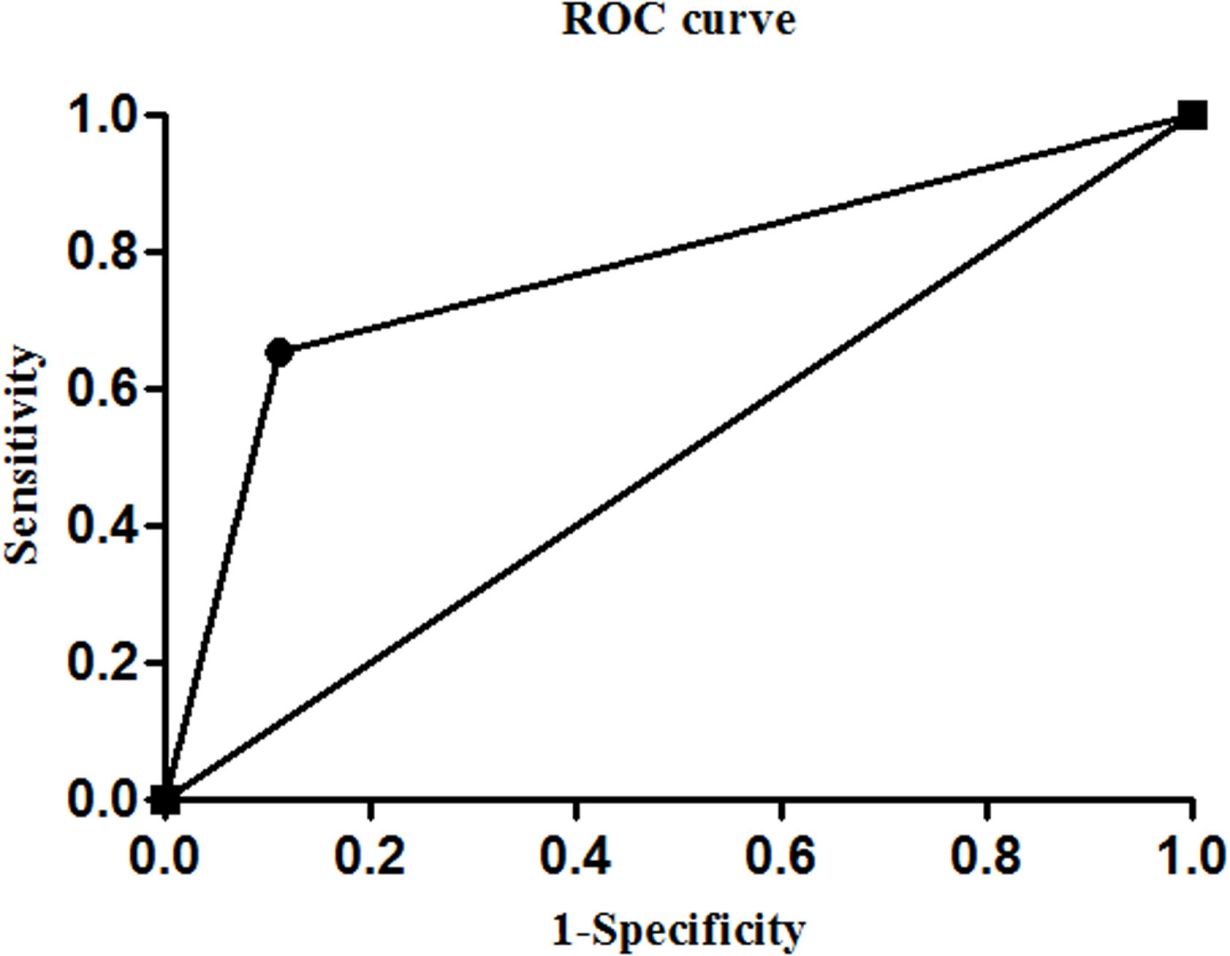
Combined detection of *Zic1* promoter methylation and the CEA level (parallel testing) in gastric intraepithelial neoplasia (GPI) plasma specimens. An ROC curve for evaluating the significance of the combined detection of these two parameters for GPI diagnosis.

In the GPI group, the sensitivity and specificity of the combined determination of the *Zic1* promoter methylation rate and CEA level (tandem testing) were 15.8% and 100.0%, respectively (Table 3). The combined assessment of these two parameters (parallel testing) had the highest sensitivity at 65.3%, with a positive predictive value reaching 98.4% (Table 3). ROC curve analysis was performed to assess the significance of the DNA methylation rate and CEA level for GPI diagnosis. The AUC for *Zic1* promoter methylation testing alone was 0.792 (S3 Fig) and that for the combined assessment of the *Zic1* promoter methylation rate and the CEA level (parallel testing) was 0.771 (Fig 4). Therefore, the detection of *Zic1* promoter methylation might be a more effective alternative for GPI diagnosis.

**Table 3:** Sensitivity and specificity of markers detected in the gastric intraepithelial neoplasia (GPI) group.

Marker	Sensitivity (%)	Specificity (%)
CEA	21.1%	88.9%
*Zic1* promoter methylation	58.4%	100.0%
CEA* *Zic1* promoter methylation	15.8%	100.0%
CEA/ *Zic1* promoter methylation	65.3%	88.9%

*: *Zic1* promoter methylation combined with the CEA level (tandem testing)

/: *Zic1* promoter methylation combined with the CEA level (parallel testing)

## Discussion

Recently, numerous genes have been found to be methylated in GC tissues, including *E-cadherin*, *RASSF1A*, *p16*, *GSTP1*, *SOCS1*, *SFRP1* and *PTEN*[17]. Reported methylation frequencies have ranged from 56% to 96%[17]. DNA methylation has also been observed in blood samples from GC patients. Several TSGs have been found to be methylated in blood samples from GC patients, including *p16*, *E-cadherin*, and *RAR*, with detection rates of 37% to 48%, respectively[18–21].

*Zic1* is aberrantly downregulated in certain types of cancer, indicative of its function as a TSG. Our previous work has revealed that the *Zic1* promoter is frequently methylated in primary gastric carcinoma tissues, with a high detection rate of 94.6%, but not in normal gastric tissues[14]. Our results have revealed that *Zic1* is a novel candidate TSG that is downregulated via promoter hypermethylation in GC. However, to the best of our knowledge, no studies have investigated plasma *Zic1* promoter methylation in GC or GIN.

In the present study, we first demonstrated that aberrant methylation of the *Zic1* promoter could be detected in plasma from GC and GIN patients at frequencies of 60.6% and 54.0%, respectively, which were significantly higher than that of the NCs. These results are consistent with those of previous studies[14]. From a diagnostic point of view, *Zic1* promoter methylation exhibited high specificity for discriminating the NCs from the GC and GIN patients (100%). Our results also demonstrated that detection of the *Zic1* promoter methylation rate had a high positive predictive value and a positive likelihood ratio and AUC for GC and GIN diagnosis, suggesting that *Zic1* promoter methylation assessment has potential utility for the diagnosis and early prediction of GC.

Many previous studies have focused on peripheral blood DNA methylation in GC rather than GIN patients, with only a handful of studies focusing on DNA methylation in GIN. The development of endoscopic techniques has allowed for the availability of more effective and less invasive treatments for EGC and GIN patients. Overall, screening for GIN contributes to the monitoring of GC and the reduction of GC-related deaths. One interesting aspect of the present study is that we demonstrated aberrant *Zic1* promoter methylation in plasma from GIN patients, with a detection rate that was significantly higher than that of the NC subjects (p < 0.001). In the future, detection of hypermethylation of the *Zic1* promoter could potentially be used as an early warning sign or to screen for GC in high-risk groups.

We also evaluated *Zic1* promoter methylation in plasma from GC patients in association with various clinicopathological parameters. No statistically significant relationships were observed between *Zic1* promoter methylation and the various clinical parameters, including tumor size, differentiation grade, lymph node status, tumor invasion depth and TNM stage; however, in our previous study[15], *Zic1* methylation has been found to be involved in GC invasiveness. In the present study, most of the GC patients were diagnosed and treated at an early stage, which may have influenced the final results. Validation in a larger population might provide insights into these differing results. Whether any correlation exists between aberrant *Zic1* promoter methylation and GC clinical characteristics requires clarification by further analysis.

CEA is a classic tumor marker that is commonly assessed in the clinic. This marker is considered to be the most frequently present in late GC patients; however, many studies have indicated that DNA methylation can be detected beginning in the early stages of GC. In our present study, an ROC curve was generated to evaluate the significance of the combined detection of the *Zic1* promoter methylation rate and CEA level (parallel testing) for the diagnosis of GC, revealing that their combined detection produced more significant results than the detection of either parameter alone. Therefore, we suggest that the combined measurement of the *Zic1* promoter methylation rate and CEA level may enhance the diagnosis of GC.

In conclusion, our results have revealed that the measurement of *Zic1* promoter methylation in plasma-derived DNA is of significance for the early detection of GC and for risk assessment in high-risk populations. The combined measurement of the *Zic1* promoter methylation rate and CEA level (parallel testing) may enhance the current guidelines for the early diagnosis of GC; however, further studies with larger sample sizes and clinical validation are required.

## Supporting Information

S1 Fig Detection of *Zic1* promoter methylation in gastric cancer (GC) plasma specimens. An ROC curve for evaluating the significance of the *Zic1* promoter methylation testing for GC diagnosis.
(TIF)

S2 Fig Combined detection of *Zic1* promoter methylation and the CEA level (tandem testing) in gastric cancer (GC) plasma specimens. An ROC curve for evaluating the significance of the combined detection of the two parameters (tandem testing) for GC diagnosis.
(TIF)

S3 Fig Detection of *Zic1* promoter methylation in gastric intraepithelial neoplasia (GPI) plasma specimens. An ROC curve for evaluating the significance of the *Zic1* promoter methylation testing for GPI diagnosis.
(TIF)
