# Supplementary figures and images for: Zic1 Promoter Hypermethylation in Plasma DNA Is a Potential Biomarker for Gastric Cancer and Intraepithelial Neoplasia
Source: PLoS One. 2015 Jul 24;10(7):e0133906. doi: 10.1371/journal.pone.0133906 (PMC4514771; doi:10.1371/journal.pone.0133906)

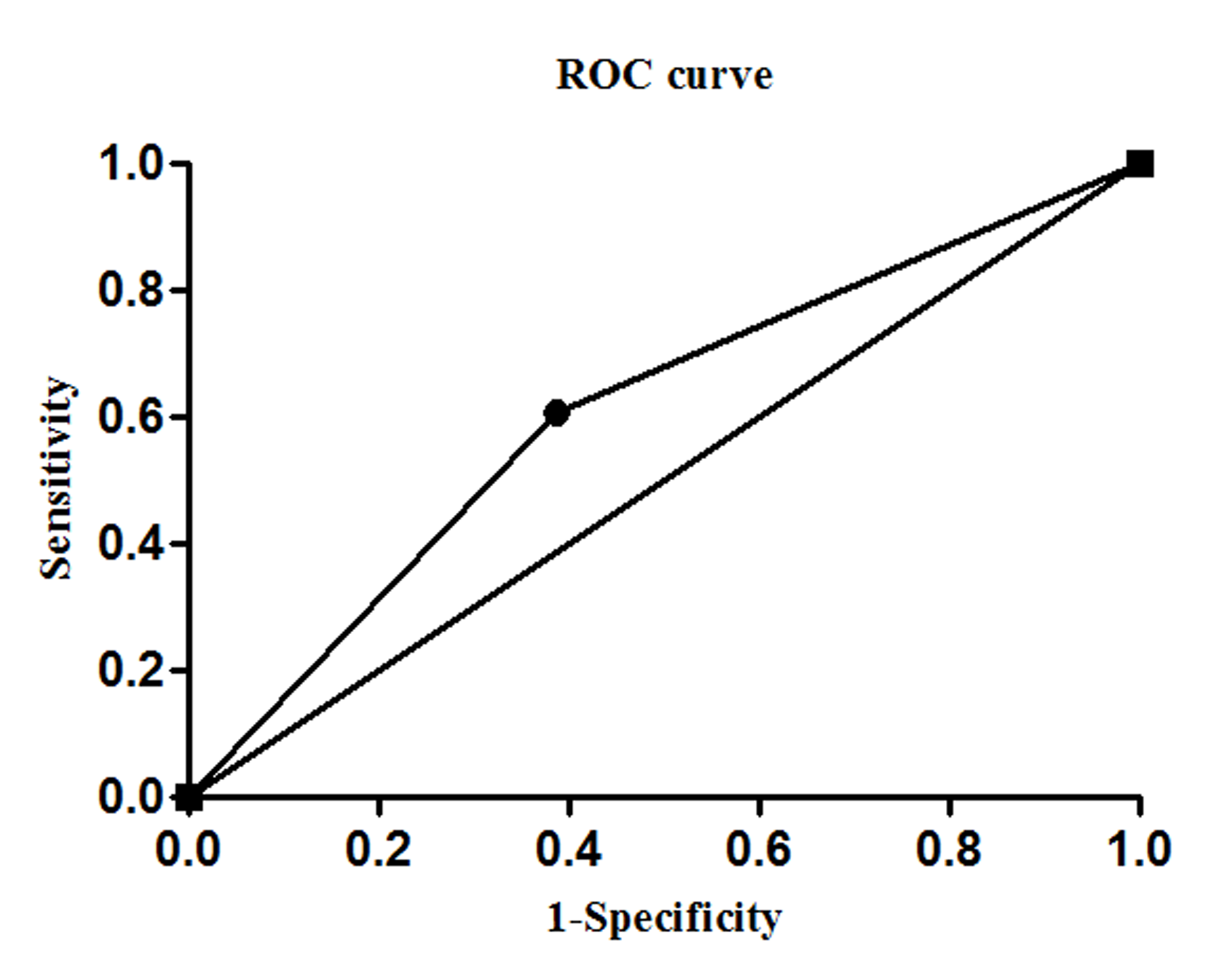

Supplement: S1 Fig — An ROC curve for evaluating the significance of the Zic1 promoter methylation testing for GC diagnosis. (TIF) [file pone.0133906.s001.tif]

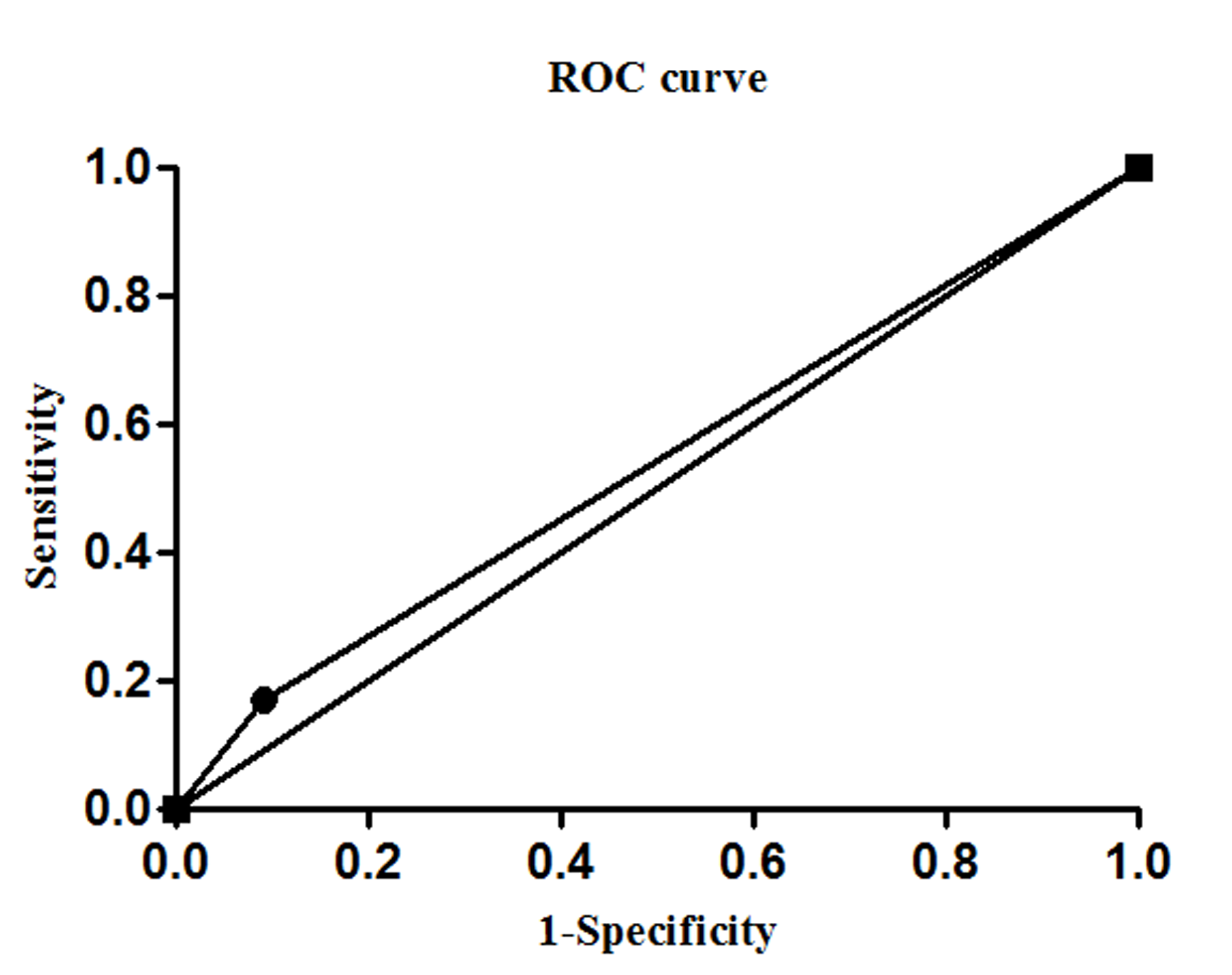

Supplement: S2 Fig — An ROC curve for evaluating the significance of the combined detection of the two parameters (tandem testing) for GC diagnosis. (TIF) [file pone.0133906.s002.tif]

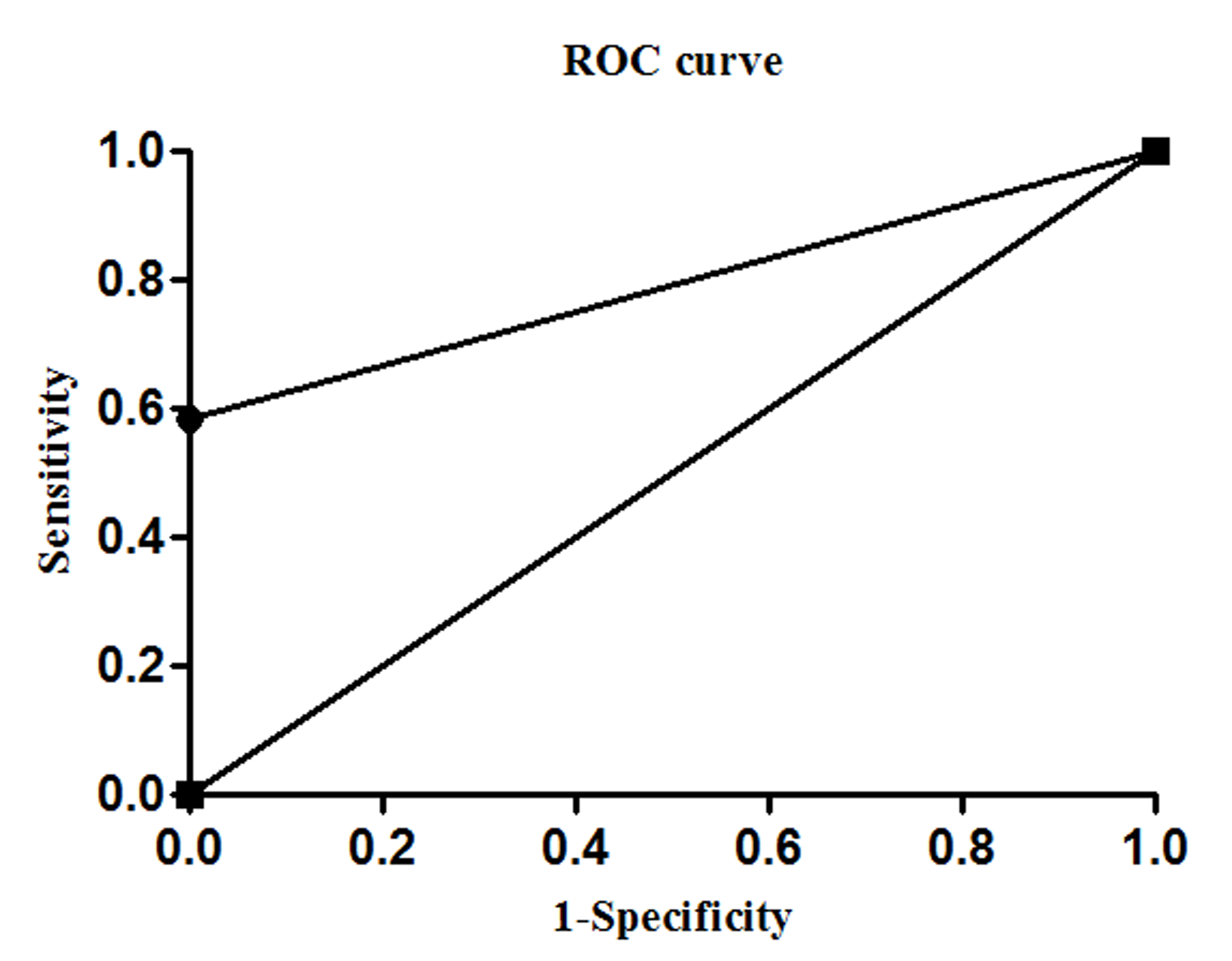

Supplement: S3 Fig — An ROC curve for evaluating the significance of the Zic1 promoter methylation testing for GPI diagnosis. (TIF) [file pone.0133906.s003.tif]
